# 

**Published:** 1959-04

**Authors:** 


					A
THE
MEDICAL JOURNAL
OE THE
SOUTH-WEST
Incorporating
The Bristol Medico-Chirurgical Journal
April 1959
VOL. 74 n0. 272 Price 4/-

				

